# Datasets on material properties and energy yields of lab-designed organic fraction of municipal solid waste (OFMSW) components

**DOI:** 10.1016/j.dib.2022.108519

**Published:** 2022-08-05

**Authors:** Gregor Sailer, Benedikt Hülsemann, Johanna Eichermüller, Florian Empl, Jens Poetsch, Stefan Pelz, Daniel Kuptz, Hans Oechsner, Joachim Müller

**Affiliations:** a University of Applied Forest Sciences Rottenburg, Schadenweilerhof, Rottenburg 72108, Germany; b State Institute of Agricultural Engineering and Bioenergy, University of Hohenheim, Garbenstrasse 9, Stuttgart 70599, Germany; c Technology and Support Centre in the Centre of Excellence for Renewable Resources (TFZ), Department of Solid Biofuels, Schulgasse 18, Straubing 94315, Germany; d Institute of Agricultural Engineering, Tropics and Subtropics Group, University of Hohenheim, Garbenstrasse 9, Stuttgart 70599, Germany

**Keywords:** Biowaste, Food and green waste, Standardized samples (recipes), Physico-chemical characteristics, Combustion, Anaerobic digestion, Hohenheim Biogas Yield Test (HBT), Aerobic and anaerobic storage

## Abstract

The organic fraction of municipal solid waste (OFMSW) is a complex material with different ingredients characterized by varying properties depending on parameters such as season or geographical region of origin. Consequently, studies on OFMSW are hard to compare due to the changing characteristics of the samples. Therefore, this article presents data on the physico-chemical composition of standardized, recipe-based OFMSW components divided into the categories “Paper”, “Green waste” and “Food waste”, and further subcategories. Data presented in this article include (1) dry matter, (2) organic dry matter, (3) C, H and N concentrations, (4) gross calorific values, (5) ash melting behavior, (6) specific biogas yield and (7) methane concentration. An application example of an experiment requiring the same starting material properties is represented by storage experiments, as performed within the original scientific article [1]. Thus, this Data in Brief article also provides additional data on recipe-based storage experiments complementing the original article. The datasets cannot only be used to estimate biowaste potentials but they can also be used for the design and execution of experiments that require standardized OFMSW samples.

## Specifications Table

**Table:** 

Subject	Waste Management and Disposal
Specific subject area	Physico-chemical characterization and energy yields of organic fraction of municipal solid waste (OFMSW) components
Type of data	25 Tables and 4 Figures
How data were acquired	Datasets for OFMSW components were acquired using standard physico-chemical analyses and instruments: • Dry matter (DM) concentration by oven drying (UNP 700, Memmert, Schwabach, Germany) according to DIN EN 13040:2007 [2] • Organic dry matter (oDM) concentration by muffle furnace (AAF 1100, Carbolite, Neuhausen, Germany) according to DIN EN 14775:2009 [3] • C, H and N concentrations by elemental analyzer (vario MACRO cube, elementar, Langenselbold, Germany) according to DIN EN ISO 16948:2015 [4] • Specific methane yield (SMY) of anaerobic digestion (AD) by Hohenheim Biogas Yield Test (HBT) with 100 mL glass syringes and CH_4_ sensor (Advanced Gasmitter, Pronova, Berlin, Germany) according to VDI 4630 [5] at 37 °C and hydraulic retention time of 35 days • Gross calorific values (GCV) by automated isoperibol calorimeter (C6000, IKA-Werke, Stauffen, Germany) according to DIN EN 14918:2009 [6] • Ash melting behavior by hot stage microscope (EM201/Ofen 1600/80, Hesse Instruments, Osterrode am Harz, Germany) according to DIN CEN/TS 15370–1:2006 [7]
Data format	Raw, processed and plotted data
Parameters for data collection	Standardized OFMSW samples were produced by blending ingredients according self-defined recipes. The samples were dried and stored until further experiments were carried out. In total, two production runs were performed. All further analyses were performed with several repetitions except for the determination of DM concentration, where the whole sample amount was dried. Based on a third production run of food waste samples, storage experiments were executed.
Description of data collection	Physico-chemical characterization of OFMSW components (DM and oDM concentrations as well as elemental compositions) including data on combustion (GCV and ash melting behavior) and on biogas as well as methane yields
Data source location	All materials were acquired in southern Germany (Rottenburg, Baden-Wuerttemberg)
Data accessibility	Repository Name: Mendeley Data DOI: 10.17632/vj6cmc4h3m.1 Direct URL to data: https://data.mendeley.com/datasets/vj6cmc4h3m/1
Related research article	Sailer et al. (2022), Improving the energetic utilization of household food waste: impact of temperature and atmosphere during storage, Waste Management, Volume 144, May 2022, p. 366–375, https://doi.org/10.1016/j.wasman.2022.04.012

## Value of the Data

• Standardized samples increase the repeatability of experiments and foster the comparability of results. The biowaste recipes in this article can be used for the design and execution of further experiments.
• This data article provides a physico-chemical characterization of standardized biowaste samples to optimize biogas plants and calculate energy potentials.
• Dataset and underlying methodical approaches are useful for biomass and bioenergy-researchers to identify individual characteristics of OFMSW subsets.
• Waste management companies and plant operators can use the presented data to optimize the energetic utilization and material use of OFMSW components.

## 1 Data Description

The organic fraction of municipal solid waste (OFMSW) is an inhomogeneous mixture of various biogenic and non-biogenic ingredients with changing physico-chemical characteristics that complicate an optimal utilization. In this data article, OFMSW components divided into different categories and subcategories are presented. All data are available online in Mendeley Data (https://data.mendeley.com/datasets/vj6cmc4h3m/1).

Based on lab-designed recipes for OFMSW, standardized samples can be generated at any time. In this way, repeatable and comparable experiments can be performed with identical starting material, which is not possible with real OFMSW samples due to their changing properties.

For each OFMSW component, data on material properties and energy yields regarding combustion behavior and anaerobic digestion (AD) are presented. All data are based on two production runs (R1 and R2) that were executed with the same procedure to determine the value fluctuations and thus the reproducibility of the recipe-based OFMSW components.

The dataset in Mendeley Data is divided into five different files. In addition to sample codes, Tables 1–3 (file A) present the recipes with ingredients for three different OFMSW categories (“Paper”, “Green waste” and “Food waste”).

In Tables 4–10 (file B), basic characteristics of OFMSW categories and subcategories are presented. Tables 4 and 5 show dry matter (DM) concentration data for the ingredients of the “Green waste” and “Food waste” category, while DM concentration data for all OFMSW subcategories are shown in Table 6. The concentrations of organic dry matter (oDM) are divided into raw data for all OFMSW subcategories (Table 7) and into mean values (Table 8). Table 9 (raw data) and Table 10 (mean values) present the concentrations of C, H and N that can also be used for energy potential calculations such as stoichiometric biogas yield or combustion potentials.

In Tables 11 and12, gross calorific value (GCV) datasets are presented while Table 13 shows data on the ash melting behavior (file C). The data of the AD experiments (biochemical CH_4_ potential tests) are shown in Tables 14 and 15 (file D). Data on both combustion and AD are only available based on the samples of R2.

Apart from the samples of R1 and R2, Tables 16–25 and Figs. 1 and 2 (file E) present raw data and additional information on storage experiments with food waste samples that were produced separately but in accordance with the recipe as presented in Table 3 (file A). For instance, the nutritional values of the food waste subcategories are presented in Table 17. In addition to the raw data, Figs. 3 and 4 show visual impressions of the storage experiments executed and presented in Sailer et al. [1].

## 2 Materials and Methods

### 2.1 Recipes of Lab-Designed OFMSW Components

The recipes were created based on sorted and weighted real OFMSW samples from own studies [1] and by considering basic data of the German Federal Statistical Office^1^ and of the German Federal Ministry of Food and Agriculture.^2^ Statistical data served as verification of the relevance of certain single ingredients chosen for the recipes. In general, the recipes contain typical products purchasable at any time in the course of the year. All ingredients for category “Food waste” were obtained in a typical supermarket and prepared (e.g., chopped) for the determination of DM concentrations. The ingredients for category “Green waste” were purchased in a typical hardware store. Both stores were located in southern Germany (Rottenburg a.N., Baden-Wuerttemberg). Further information on the recipes can be found in the research article [1].

The OFMSW recipes (Tables 1–3) present the proportion of each ingredient in each subcategory mixed as fresh mass (FM). In addition, the DM concentration of each substance of the recipes was measured.^3^ The OFMSW samples were prepared in two consecutive runs (R1 and R2) in accordance with recipes presented in Tables 1–3 (file A).

### 2.2 Dry Matter and Organic Dry Matter Concentration

DM concentration was determined by drying the whole substance (e.g., banana peel and fruit pulp) at 105 °C in a drying oven (UNP 700, Memmert, Schwabach, Germany) for at least 24 h until constant weight. This procedure was in accordance with standard procedures (DIN EN 13040:2007 [2]) mentioned in the German Biowaste Ordinance.^4^ A few ingredients of category “Food waste” of R2 were not completely dry due to compact storage in the drying vessels. Therefore, the subcategory mixtures (containing all ingredients) were additionally dried in R2 to ensure a dry state.

All samples were milled to particle sizes of approx. 1 mm, either with a cutting mill (Pulverisette 19, Fritsch, Idar-Oberstein, Germany) or with a customary mixer (WMF Kult Pro 1400 W, WMF Group, Geislingen-Steige, Germany). The samples of category “Green waste” (Table 2) were processed in the cutting mill. The samples of category “Food waste” (Table 3) and category “Paper” (Table 1) were milled in the mixer. All processed laboratory samples were stored in dry state until further experiments were carried out. Before further chemical analyses, the laboratory samples were dried repeatedly (105 °C) for approx. 2 h to ensure a dry condition for each sample.

The data of oDM concentrations were determined through combustion in a muffle furnace (AAF 1100, Carbolite, Neuhausen, Germany) in accordance with DIN EN 14775:2009 [3] by using approx. 1 g of dry material in a ceramic crucible.

### 2.3 Elemental Analysis

The analysis of C, H and N concentrations was carried out with an elemental analyzer (vario MACRO cube, elementar, Langenselbold, Germany) according to DIN EN ISO 16948:2015 [4] with approx. 40 mg of dry material per sample pressed into a zinc foil coated tablet. S was not measured simultaneously in favor of the measurement accuracy of C, H and N. No combustion additives were used. Data for C, H and N can be used for the calculation of stoichiometric biogas or CH_4_ yields [5]. The concentration of O (m/m_DM_ in%) can be calculated based on the concentrations of C, H, N and ash.

### 2.4 Gross Calorific Values and Ash Melting Behavior

The GCV was determined with approx. 1 g of dry material per sample of R2 at a constant volume with an automated isoperibol calorimeter (C6000, IKA-Werke, Stauffen, Germany). This procedure was in accordance with DIN EN 14918:2009 [6]. Based on the GCV data, the net calorific value can be calculated at a constant volume. The ash melting behavior was measured in duplicate for samples of R2 according to DIN CEN/TS 15370–1:2006 [7]. Therefore, ash of each OFMSW component sample was prepared [3] and combined with C_2_H_5_OH (96°%). The mixture was then milled in a mortar and pressed into a cylinder for measurement in a hot stage microscope (EM201/Ofen 1600/80, Hesse Instruments, Osterrode am Harz, Germany).

### 2.5 Anaerobic Digestion Experiments

The AD data for samples of R2 were acquired based on the Hohenheim Biogas Yield Test (HBT), which is a batch method to determine the specific methane yield (SMY) of biogas substrates. The experiments were operated for 35 days at mesophilic temperature (37 °C). The digesters were filled with approx. 30 g_FM_ of inoculum (own digestate from a 400 L laboratory reactor with a low organic loading rate and a balanced nutrient supply) together with approx. 0.4 g_DM_ per OFMSW component to achieve an inoculum/substrate ratio of 2:1 based on oDM. The blank variants were filled with approx. 50 g_FM_ of inoculum. In addition, reference samples were used to verify the quality of the AD experiments. The gas yields of the inoculum were deducted from the variants consisting of inoculum and OFMSW samples. All SMY data were calculated for standard conditions (0 °C and 1013 hPa, dry gas). The whole experiment setup and processing was done according to VDI 4630 [5]. With the presented data for oDM and DM concentrations of all OFMSW components, a re-calculation of AD yields to different reference units is possible. Detailed proceedings on the HBT method can be found in Helffrich and Oechsner [8] and in Mittweg et al. [9]. Further information on the 400 L laboratory reactor and the digestate composition is presented in Hülsemann et al. [10].

### 2.6 Storage Experiments As An Application Example For Recipe-Based OFMSW Components

Separately from the production and analysis of the OFMSW components of R1 and R2, storage experiments under aerobic and anaerobic conditions and for different temperatures (5 °C and 20 °C) have been executed based on recipe of category “Food waste” as presented in Table 3. Therefore, food waste samples based on a mixture of 100 g_FM_ per subcategory with the proportion as displayed in Table 3 have been generated. Thus, the storage experiments were executed with 500 g_FM_ per sample. All products used for the recipe-based generation of food waste samples were aliments, which are characterized by natural variations (e.g., considering water contents). Therefore, the recipe comprised a broad variety of food products. It was assumed that the mixture balanced the natural variations of each single product.

In the course of the experiments, FM, DM, oDM, GCV and elemental compositions (C, H, N) were measured with methods as described above. The GCV was only measured for the samples stored at 20 °C for day of experiment (DOE) 0, 4, 8, 12, 16 and 20 as well as for the respective anaerobic sample. This procedure was chosen due to the assumption of a higher biologic activity at 20 °C. Both GCV and stoichiometric energy potentials (calculated based on C, H and N) were used to estimate energy potential losses of the food waste samples during storage.

Prior to the analyses, the whole food waste sample amount was ground for approx. 60 s to particle sizes of approx. 1 mm with a customary mixer (WMF Kult Pro 1400 W, WMF Group, Geislingen-Steige, Germany) to generate laboratory samples. The laboratory samples were then stored for several days in dry state until further analyses were carried out. To ensure a dry state, all samples were additionally dried for 1 h at 105 °C prior to each analysis. In addition, the samples were homogenized before each experiment.

Data on the storage experiments are presented in Tables 16–25 and in Figs. 1–4 (file E). Detailed descriptions of the storage experiments and a scientific discussion can be found in the original article [1]. This article supplements the primary research article by providing raw data on the analyses and by delivering additional data regarding the storage experiments.

## Ethics Statement

The authors declare that they have followed the rules of scientific research and publishing. No conflict of interest exists in this study.

## CRediT authorship contribution statement

**Gregor Sailer:** Conceptualization, Methodology, Validation, Formal analysis, Investigation, Visualization, Writing – original draft, Writing – review & editing. **Benedikt Hülsemann:** Methodology, Validation, Investigation, Writing – original draft. **Johanna Eichermüller:** Conceptualization, Investigation. **Florian Empl:** Writing – original draft. **Jens Poetsch:** Conceptualization, Supervision. **Stefan Pelz:** Conceptualization, Project administration, Funding acquisition, Supervision. **Daniel Kuptz:** Validation, Writing – original draft. **Hans Oechsner:** Writing – review & editing, Supervision. **Joachim Müller:** Conceptualization, Methodology, Validation, Formal analysis, Visualization, Writing – original draft, Writing – review & editing, Supervision.

## Declaration of Competing Interest

The authors declare that they have no known competing financial interests or personal relationships, which have or could be perceived to have influenced the work reported in this article.

## Data Availability

Datasets on material properties and energy yields of lab-designed organic fraction of municipal solid waste (OFMSW) components (Original data) (Mendeley Data).

## Appendix

The following files can be found online at Mendeley Data: **(A)** Recipes for categories and subcategories of OFMSW (Tables 1–3). **(B)** Basic characteristics of categories, subcategories and ingredients of OFMSW (Tables 4–10). **(C)** Energy yields (combustion) of categories, subcategories and ingredients of OFMSW (Tables 11–13). **(D)** Energy yields (anaerobic digestion) of categories, subcategories and ingredients of OFMSW (Tables 14–15). **(E)** Storage experiments with food waste samples (Tables 16–25, Figs. 1–4).

## Footnotes

1 German Federal Statistical Office (Destatis), 2018, Statistical Yearbook 2018, accessible under https://www.destatis.de/DE/Themen/Querschnitt/Jahrbuch/statistisches-jahrbuch-2018-dl.html (21.04.2022).

2 German Federal Ministry of Food and Agriculture (BMEL), 2017, Nutrition Report 2018, accessible under https://www.bmel.de/SharedDocs/Downloads/DE/Broschueren/Ernaehrungsreport2018.html (21.04.2022).

3 The DM-based proportion of each single substance differs for most of the recipes. However, a DM based production is also possible but has to be calculated based on DM concentration values, the desired sample amount and the proportion in each subcategory.

4 *German Biowaste Ordinance (BioAbfV), 2017, Regulation on the reclamation of bio waste of agricultural, silvicultural or horticultural soils, accessible under*https://www.gesetze-im-internet.de/bioabfv/BioAbfV.pdf (21.04.2022).
